# Were SARS-CoV-2 self-tests used for their intended purpose? The experience in Belgium

**DOI:** 10.1186/s12913-023-09704-0

**Published:** 2023-06-29

**Authors:** Yves Lafort, Laura Cornelissen, Dieter Van Cauteren, Barbara Verboven, Sabine Drieskens, Simon Couvreur, Lize Hermans, Koen Straetmans, Tinne Lernout

**Affiliations:** 1 Department of Epidemiology and Public Health, Sciensano, Brussels, Belgium; 2 Association of Pharmacists Belgium, Brussels, Belgium

**Keywords:** SARS-CoV-2 testing, Self-testing, Belgium

## Abstract

**Background:**

Self-testing has been promoted as a means of increasing COVID-19 test coverage. In Belgium, self-testing was recommended as a complement to the formal, provider-administered indications, such as out of courtesy before meeting others and when feared to be infected. More than a year after the introduction of self-testing their place in the test strategy was evaluated.

**Methods:**

We assessed trends in the number of self-tests sold, the number of positive self-tests reported, the proportion sold self-tests/total tests, and the proportion of all positive tests that were confirmed self-tests. To evaluate the reason why people use self-tests, we used the results of two online surveys among members of the general population: one among 27,397 people, held in April 2021, and one among 22,354 people, held in December 2021.

**Results:**

The use of self-tests became substantial from end 2021 onwards. In the period mid-November 2021 – end-of-June 2022, the average proportion of reported sold self-tests to all COVID-19 tests was 37% and 14% of all positive tests were positive self-tests. In both surveys, the main reported reasons for using a self-test were having symptoms (34% of users in April 2021 and 31% in December 2021) and after a risk contact (27% in both April and December). Moreover, the number of self-tests sold, and the number of positive self-tests reported closely followed the same trend as the provider-administered tests in symptomatic people and high risk-contacts, which reinforces the hypothesis that they were mainly used for these two indications.

**Conclusions:**

From end 2021 onwards, self-testing covered a significant part of COVID-19 testing in Belgium, which increased without doubt the testing coverage. However, the available data seem to indicate that self-testing was mostly used for indications outside of official recommendations. If and how this affected the control of the epidemic remains unknown.

## Background

The coronavirus disease 2019 (COVID-19) pandemic has caused several major waves of infection around the world in the past years [1]. To contain the epidemics, most Western countries applied, especially during the first waves, a test and trace strategy. This consisted of (i) rapid confirmation and isolation of suspected clinical cases and (ii) tracing, quarantining and testing of their contacts [2, 3]. In addition, screening testing of asymptomatic persons was used in specific situations, such as for arriving travelers or before visiting vulnerable people. Initially, testing was only performed with nucleic acid amplification tests (NAATs), primarily using reverse-transcriptase polymerase chain reaction (RT-PCR). However, these tests require specialized laboratory equipment, are expensive, and results are usually not available until the next day or even later. In Belgium, for example, the median turnaround time between sampling and reporting during peak periods in the first COVID-19 year was sometimes up to two days or more [4]. Therefore, during 2020, alternative tests were developed for the detection of SARS-CoV-2 antigens using immune-chromatographic techniques, which provide results in less than 20 min and can be used at the point-of-care [5, 6]. Beginning 2021, several of these rapid antigen tests (RATs) entered the market as self-tests (STs), in which an unqualified user could self-sample and autonomously test a specimen, usually nasal. This was in addition to the tests administered by qualified providers such as pharmacists, general practitioners and lab technicians (referred to as ‘provider-administered’ tests in the remaining of the article). Several countries developed guidelines for the use of STs, weighing the benefits against the risks.

In April 2021, STs became available in Belgium in pharmacies initially for €8/piece, with later a possibility to reduce it to €5 (with a subsidized price of €1 for the most vulnerable third of the population). Guidelines were developed listing the, initially limited, indications for home self-testing [7]. Two main indications were retained: (i) out of courtesy, to avoid infecting others, before contacting people outside the household and when one feared that even by taking precautions there is still a risk of transmission; (ii) to ensure that one is not infected after a situation that is not classified as an official high-risk contact, but in which one feared to be infected. If the test was negative, all precautionary measures still had to be observed, and positive STs always had to be confirmed with an RT-PCR test (or with a provider-administered RAT from November 2021 onwards). It was emphasized that self-testing could never replace test indications for symptomatic patients that fulfilled the case definition of a possible COVID-19 case, high-risk contacts (HRC), or incoming travelers for whom testing was mandatory. Testing for these indications, using RT-PCR or RAT, was freely available at testing centra, general practitioner practices, hospital emergency services and pharmacies (RAT only). From July 1, 2021, STs were also sold in supermarkets, in addition to pharmacies, at ~€2–6.

**Fig. 1:**
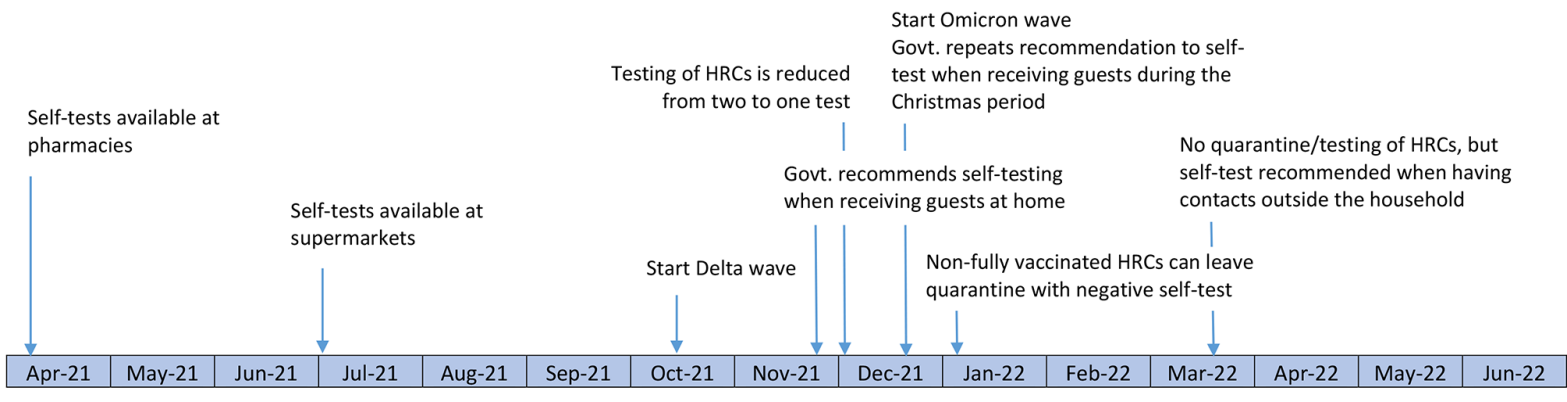
Timeline of key events in the strategy on the use of COVID-19 self-tests in Belgium

In November 2021, testing capacity was overloaded by a new wave of infections caused by the Delta variant, followed in January 2022 by a first Omicron wave. The weekly number of provider-administered tests exceeded 800,000 in some weeks of this period, compared with an average of about 300,000 per week in the January-September 2021 period. In this context, a broader use of self-testing was recommended when hosting guests in private settings (e.g. in the Christmas period). From January 2022 onwards quarantine measures and provider-testing for high-risk contacts were progressively lifted and self-testing was recommended after a high-risk contact. First, negative self-testing was required for ending quarantine in non-fully vaccinated individuals. In March all quarantine and test measures for HRCs were stopped, and a self-test was recommended before meeting people outside the household. The timeline of key changes is presented in Fig. 1.

**Fig. 2:**
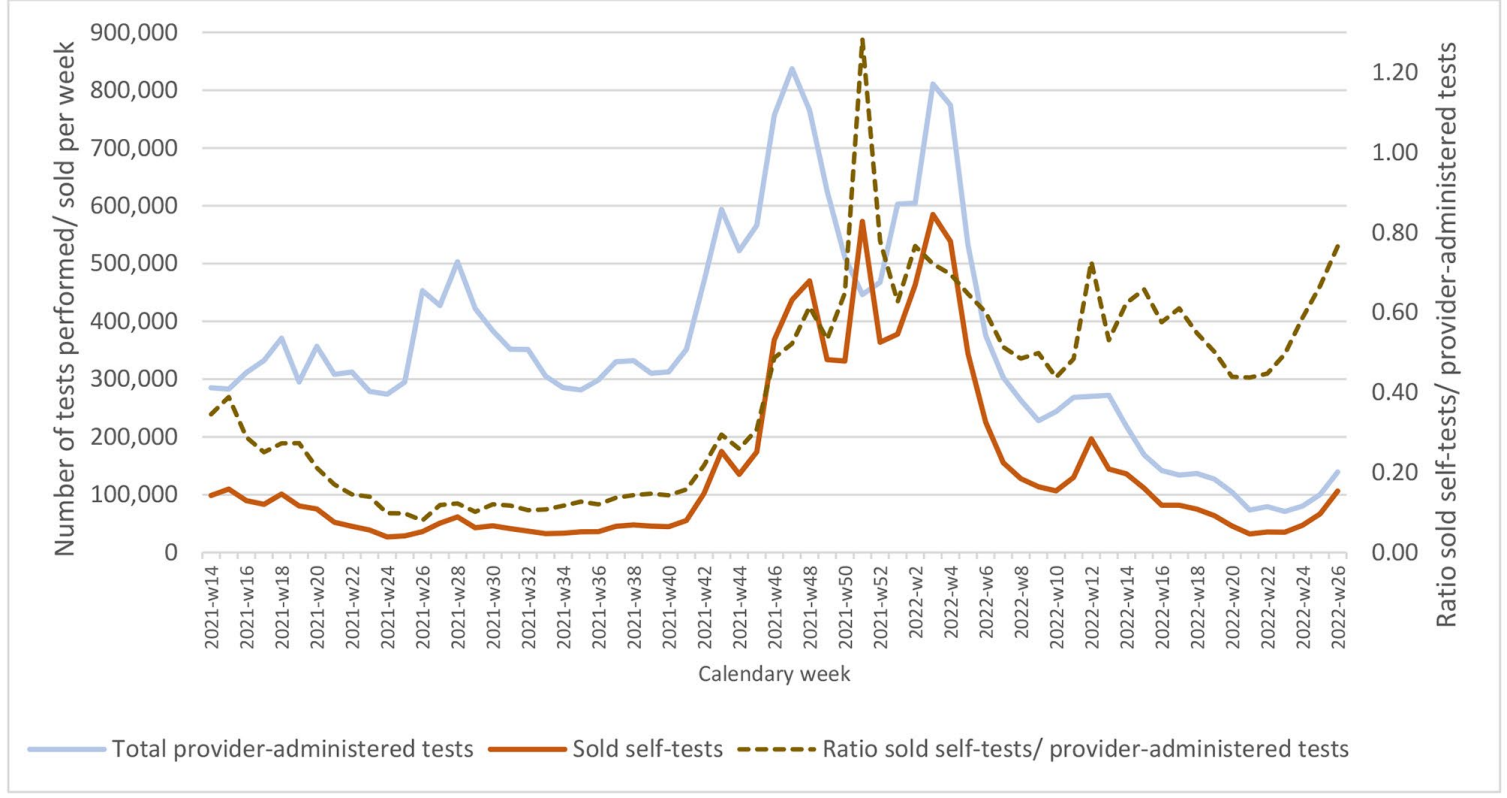
Evolution of the weekly number of registered self-tests sold at pharmacies, compared to the number of provider-administered tests, April 2021-June 2022

**Fig. 3:**
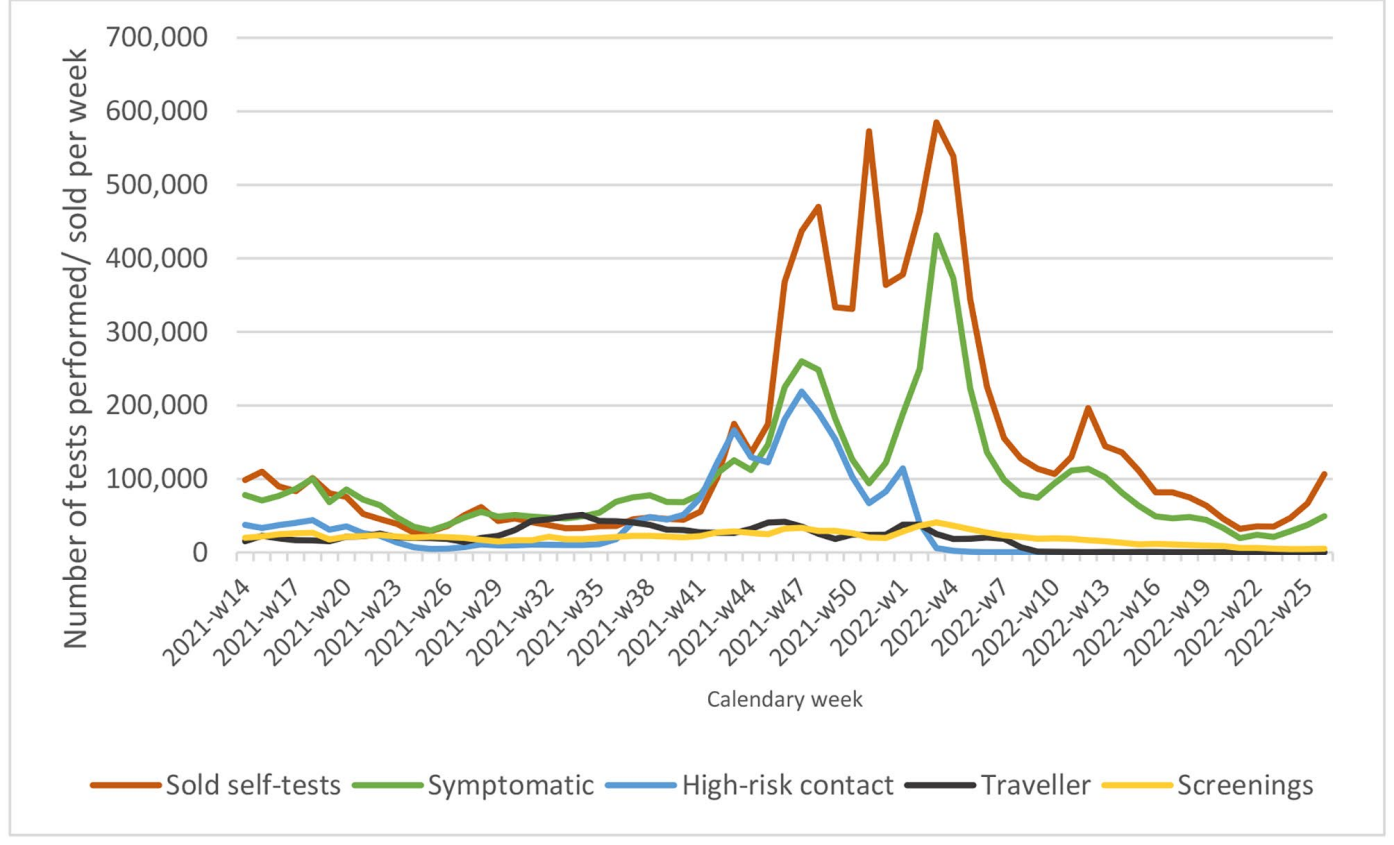
Evolution of the weekly number of sold self-tests, compared to the number of provider-administered tests per indication, April 2021-June 2022

Over time, feedback from health care providers and the general public suggested that STs were being primarily used for indications for which they were not intended, especially when having COVID-like symptoms. In general, literature has focused mostly on evaluating the technical aspects of different testing methods. Less is known about testing strategies as a whole, involving also behavioral and contextual factors. More than a year after the introduction of STs for COVID-19 in Belgium, it was therefore considered useful to evaluate the extent of self-testing and the reasons why people use it.

## Methods

We used data available from the routine national surveillance systems, complemented with information on patients’ attitudes reported by different surveys. This included (i) the weekly number of STs sold at pharmacies; (ii) the weekly number of reported positive STs, confirmatory tests and positive confirmatory tests; (iii) the results of an online survey by the Association of Pharmacists Belgium (APB); and (iv) the results of the 9th COVID-19 health survey conducted by the Belgian Institute of Public Health (Sciensano) among adult Belgian residents.

All pharmacies in Belgium are requested to report the daily number of sold STs to the APB, on a weekly basis. Reporting is mandatory for STs sold to people who qualify for reduced pricing of the tests, and voluntary for the other STs sold. We assessed the evolution of the number of weekly sold STs from the start until July 3, 2022. To evaluate the relative importance of self-testing within the test strategy, we calculated (per week) the proportion of sold STs among all tests (sold STs plus provider-administered tests).

The Belgian surveillance system does not include the reporting of the result of each self-test performed by a citizen. According to the official recommendations, only positive STs must be reported and confirmed by a provider-administered test, but the adherence to this recommendation is not known. We used the number of reported positive STs, the number of confirmatory tests and the number of confirmatory tests that tested positive. We assessed the evolution of the number of weekly confirmatory tests until July 3, 2022 and calculated the proportion of all positive tests that were confirmed positive STs. We also calculated the ratio confirmed positive STs/sold STs.

APB contracted a research company (DayOne) that conducted, in April 2021, an online survey among adult Belgian residents ( > = 18 years) to explore their perception on self-test use. The company used their existing database of a representative sample of Belgian adults and did not recruit specifically for the self-testing survey. Recruitment started one week after introduction of the STs in pharmacies, and continued until 2000 people who reported to have ever purchased a self-test were reached. A total of 27,397 people participated. We used from this survey in our evaluation the proportion of participants who ever bought/used a self-test, the reason for not buying a self-test, the reason for buying/using a self-test, the appreciation of the price, the easiness of the test, the result of the test, and the conduct when the test is positive/negative.

In a series of periodic online COVID-19 health surveys of a sample of adult Belgian residents (18 years and older), Sciensano included a number of questions about COVID-19 self-testing in the 9th survey, conducted in December 2021 [8]. A mixed sampling approach was applied, combining river sampling trough Sciensano, press, local community organizations, health insurance funds, elderly organizations, sports federations, higher education institutes and young adult clubs; recruitment of participants in previous COVID-19 health surveys via e-mail; and snowball sampling via participants and Sciensano employees. A total of 22,354 individuals participated. For our evaluation we used the proportion who ever used a self-test, the number of STs used, the reason for using a self-test, the result of the self-test and whether positive STs had been confirmed with PCR. Proportions were weighted for age, gender, province, and level of education.

## Results

### Number of sold self-tests

Figure 2 presents the evolution of the number of registered STs sold at pharmacies, compared with the number of provider-administered tests (PCR + RAT) between 5 April 2021 and 3 July 2022. Overall, the number of sold STs followed the same evolution as the provider-administered tests. Initially, (April 5 – October 17, 2021) the weekly number of sold STs was relatively low (average of 54,429 STs sold/week; proportion sold STs/all tests = 14%). The number and proportion increased sharply in the second half of October 2021, at the start of the Delta wave, to then follow roughly again the same trend as the Provider-administered tests, at a proportion of around 37%. The average number of sold STs/week was the highest in the period of November 15, 2021 – February 6, 2022 (432,134/week). Both the absolute number of tests sold and the relative importance of STs in the total amount of testing shows a marked peak in the week before Christmas (572,817/week, proportion 56%). Numbers then reduced to a low of 34,277/week in the period May 23 - June 12, 2022. Since the end of May the proportion appeared to rise again and was 43% in the week of June 27-July 3, 2022.

Figure 3 shows the trend of the number of sold STs compared with the number of Provider-administered tests for the four main indications: symptomatic, high-risk contact, arriving traveler and various screenings. Except for the peak in the week before Christmas, the trend of STs sold in 2021 closely follows the same trend as symptomatic and high-risk contact testing, while this trend is not observed for the other indications. In 2022, sold STs follow the same trend as symptomatic testing, but no longer of high-risk contact testing because changes in testing strategy for this indication.

#### Number of reported positive self-tests

**Fig. 4:**
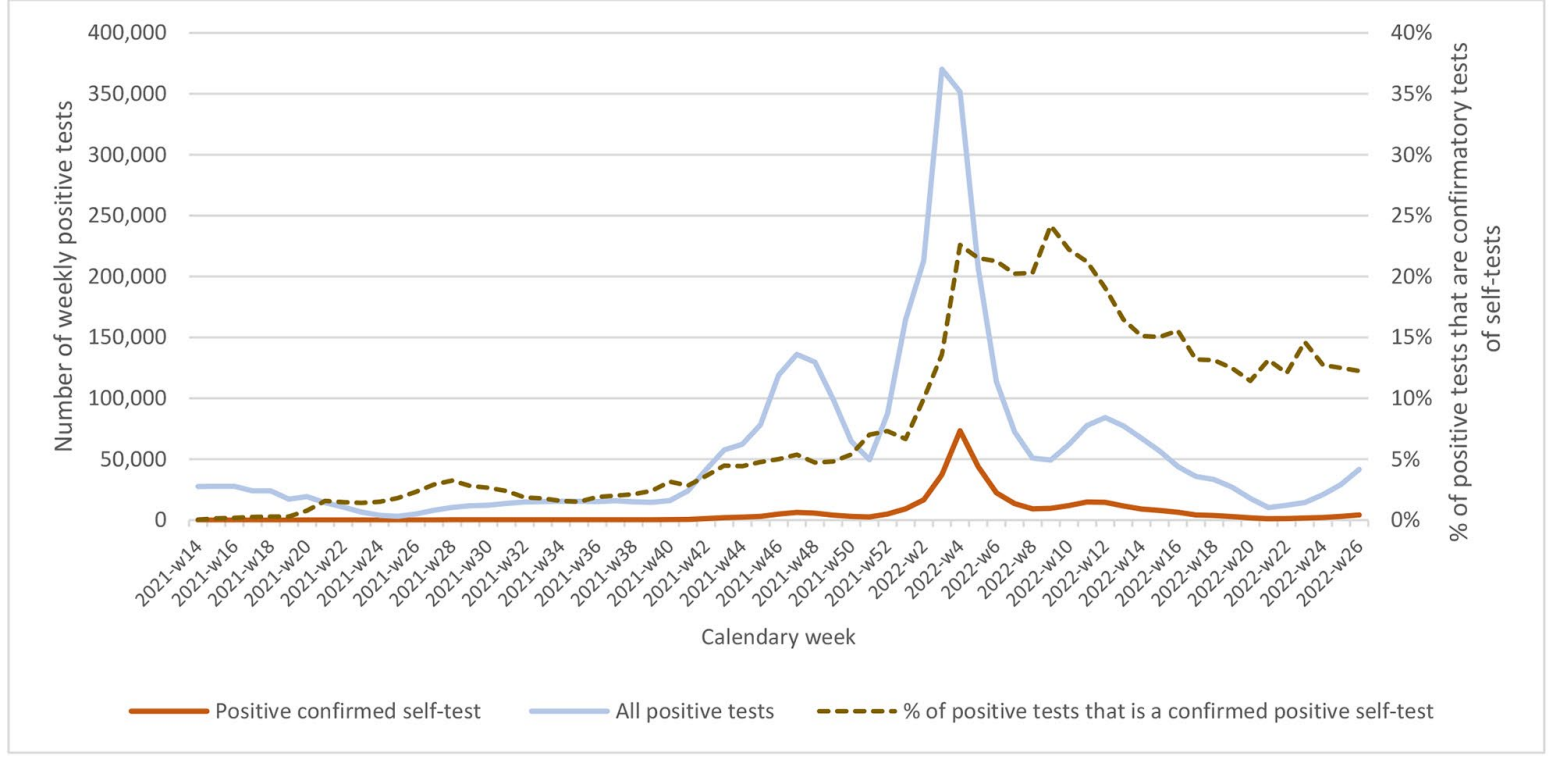
Evolution of the number of confirmed positive self-tests, compared to the total number of positive tests, April 2021-June 2022

By July 3, 2022, a total of 437,368 positive STs had been reported and 431,232 confirmatory tests had been performed (99%). Of these, 384,301 (89%) had a positive confirmatory result, a percentage that remained quite constant over time. Figure 4 presents the trend in the number of positive results for the confirmation of a positive self-test. Compared with the total number positive tests it shows a similar trend. As with the number of tests performed, it largely followed the same trend as symptomatic and high-risk contact positive testing in 2021, and the same trend as symptomatic testing in 2022, and less the trends of other test indications. The number of confirmed tests remained relatively low until October 2021, with an average of 169 tests/week. It then sharply increased to reach a peak of 73,360 in the week between Christmas and New Year. It declined to a low of 1,276 tests in the first week of June, with a small intermediate peak in the week of 14–20 March (14,817 tests) and increased again to 4,287 tests/week at the beginning of July 2022.

Initially, positive self-test confirmatory tests represented only a small percentage of all positive tests (1.2% in the period April-September 2021). From October 2021 onwards, at the start of the Delta wave its share started to slowly increase with a sharp increase in January 2022 to reach a peak of 22.6% in the last week of January. This coincided with the Omicron wave and important reductions in the indications for provider-administered tests. It then declined slightly and increased again to another peak of 24.2% in the first week of March 2022. Since then, its share decreased and was on average 12.0% in the period April 25-July 3, 2022.

**Fig. 5:**
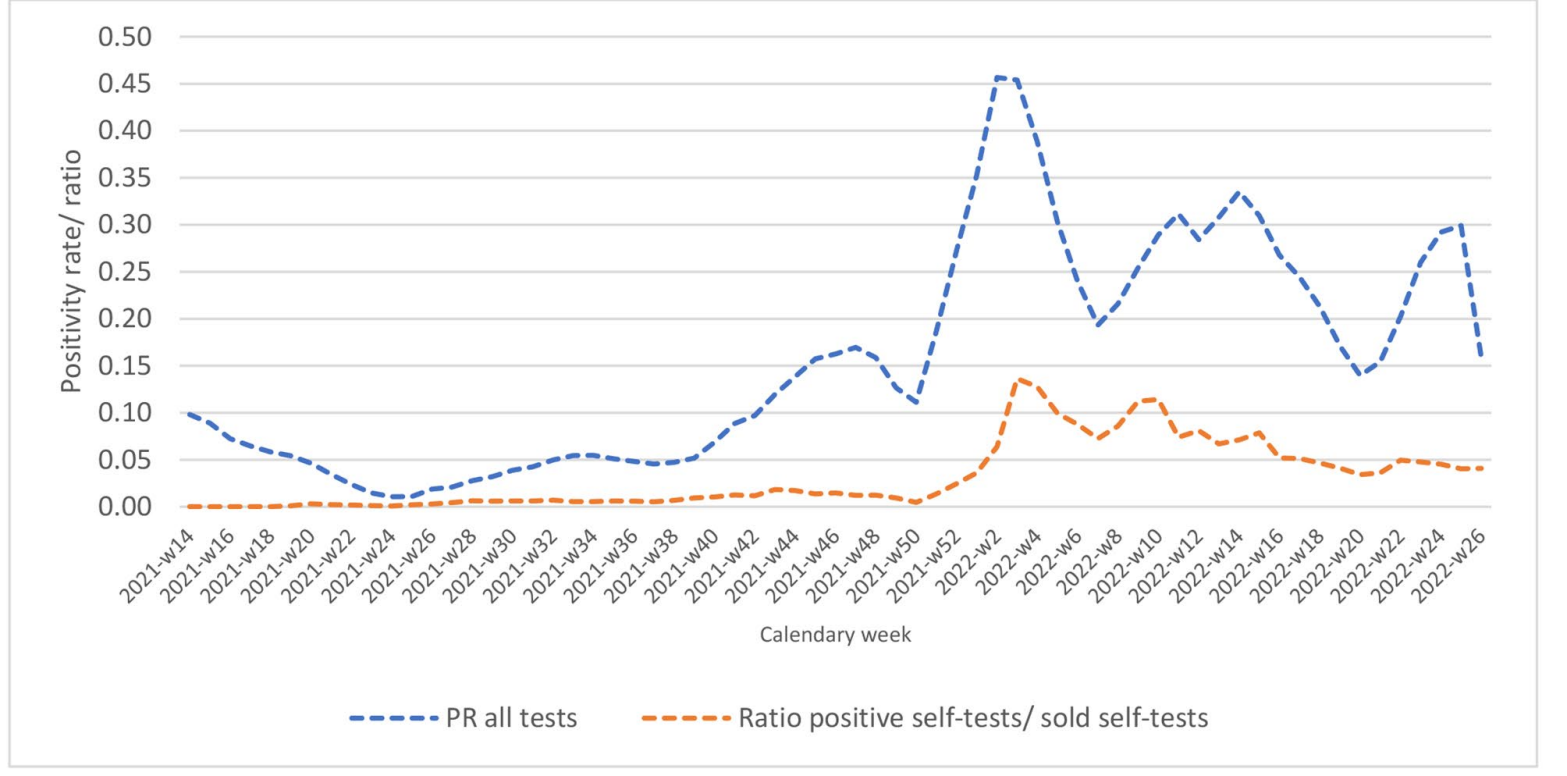
Evolution of the ratio of reported positive self-tests over the number of sold tests, compared to the overall test positivity rate, April 2021-June 2022

Using the ratio of reported positive STs over the number of sold tests as a rough proxy for the positivity rate of STs, we observe that it follows the same trend as the overall test positivity rate (Fig. 5). It peaked to 0.14 in the last week of January 2022 and again to 0.11 in the week of 14–20 March 2022. In contrast, from April 25-July 3, 2022, it was on average 0.04.

The key results of the APB survey are shown in Table 1. In total 27,397 people participated, of whom 7.3% reported having purchased a self-test and 4.1% having used it. The most reported reasons for using a test were having symptoms (34.0%) and having had a risk contact (26.9%). Similarly, not having symptoms was a very commonly cited reason for not having purchased a test (in 40.4%) or not having used a test bought (46.4%). Of those who had purchased a self-test, about half (51.5%) thought the price was too high. Of those who had used a self-test, a minority (5.9%) found it more difficult than expected. 17% of the STs performed had a positive result. The majority (86.1%) were aware that a confirmation test must be requested in the event of a positive test result. About one in four (27.4%) did not appear to know that in the event of a negative result, all preventive measures must still be followed.

(Table 1 Results of the online survey carried out by APB among 2000 people who ever purchased a self-test, Belgium, April 2021 (n = 27 397))

Table 2 presents the results of the 9th COVID-19 health survey, conducted 8 months after the APB survey, in December 2021. After weighing for age, gender, province, and level of education, almost half (45.1%) of Belgians older than 18 years reported at that time to ever have used a self-test, with an average of 3.2 tests. As in the APB survey, main reasons for testing were having symptoms (31.4%) or after a risk contact (26.9%). About 7% of the STs was positive and for 81.8% of those tests a confirmatory PCR test was performed.

**Table 1:** Results of the online survey carried out by APB among 2000 people who ever purchased a self-test, Belgium, April 2021 (n = 27 397)

Question	%
Ever purchased a self-test	7.3%
**People who reported to have ever purchased a self-test (N = 2000)**	
*For whom was self-test purchased*	
For private use	99.4%
For employees	0.7%
*Appreciation of price*	
Too high	51.5%
Normal	47.2%
Too low	1.4%
*Was the self-test used at the time of interview*	
Yes	56.2%
No	43.9%
*What to do when the test is positive*	
Nothing	1.9%
Be prudent (avoid people at risk)	0.7%
Stay home in isolation	11.3%
Contact my doctor/ test center for a confirmatory test	86.1%
*What does a negative test means*	
100% sure not to be infected	9.6%
Highly likely not to be infected, preventive measures not necessary	17.8%
Probably not infected, but preventive measures still necessary	72.6%
**People who reported to have used the self-test (N = 1123)**	
*Reason for having used the self-test*	
Symptoms	34.0%
Risk contact	26.9%
Regular self-testing	26.5%
In the context of a job	17.5%
Required for an event	3.4%
*Ease of use*	
Easier than expected	43.0%
As expected	51.1%
Harder than expected	5.9%
*Test result*	
Positive	17.0%
negative	83.0%
**People who reported to have purchased, but not yet used the self-test (N = 877)**	
*Reason for purchasing the self-test, if not yet used*	
In case of having symptoms	46.4%
In case of having a risk contact	27.0%
For a special occasion, for example before visiting someone	49.7%
Still doubt about the use	3.0%
**People who reported never having purchased a self-test (N = 25,397)**	
*Reason why no self-test purchased*	
No symptoms	40.4%
Vaccinated	20.1%
No need, because respecting all preventive measures	19.4%
Don’t see the point of it	14.9%
Don’t believe they are accurate	14.6%
Plan to purchase one in the near future	6.1%
Heard on the news/ read in the newspaper that it isn’t useful	5.2%
Heard on the news/ read in the newspaper that there weren’t enough	5.1%
Had corona in the past 90 days	3.9%
Didn’t know it was possible	3.6%
Difficult to get to a pharmacy, supermarket would be more convenient	1.2%

**Table 2:** Results of the online survey carried out by Sciensano, Belgium, December 2021 (N = 22,345),

Question	n	Weighted %
Ever used a self-test (N = 18,547)	8,365	45.1%
**People who reported to have ever used a self-test (N = 8,996)**		
Average number of self-tests used	3.2	
*Reason for using a self-test (N = 8,974)*		
Because having symptoms	2,821	31.4%
After a risk contact in which feared to have been infected	2,415	26.9%
Before visiting someone	1,915	21.3%
Before going to work/school	638	7.1%
Before or after travel	423	4.7%
Having been altered for a risk contact by the Coronalert app	242	2.7%
Other	520	5.8%
*Result of last test*		
Positive	621	6.9%
Negative	8,360	92.9%
Invalid/failed	18	0.2%
**People who reported a positive result of the last test (N = 613)**		
*Confirmation of positive self-test*		
Yes	502	81.8%

## Discussion

To our knowledge, this is the first evaluation to examine the extent to which and why people are using SARS-CoV-2 self-tests on a national scale. We used the number of reported STs sold at pharmacies as a proxy for the total number of used STs. This is most certainly an underestimation because the reporting was not mandatory and STs were also sold in supermarkets from July 1st, 2022. No figures are collected on the number of STs sold at supermarkets and we can assume that they represent an important number because of the relatively lower prices and barriers compared to tests sold at pharmacies. We also cannot rule out that the ratio of tests sold in supermarkets/pharmacies changed over time. However, we believe that the ratio remained fairly constant. At the time when STs increased in use, they had been available in supermarkets for a long time and people were familiar with both options for buying STs. Our proxy therefore allows detecting trends.

The share of STs in overall testing increased sharply in the last months of 2021. The proportion of all tests that were reported sold STs since that period was 37%. We can therefore conclude that they represented a high share of all testing, especially considering it being an underestimate. The increase coincided with the beginning of the Delta wave and stronger government awareness. We observed a peak in the use of STs during the Christmas period, probably reflecting the government’s strong recommendation to take a self-test before participating in the festivities around Christmas and New Year.

We calculated the percentage of all positive tests that were confirmatory tests of positive STs to estimate the share of self-testing in detecting COVID-19 cases. However, not all people register or confirm their positive self-test. Indeed, in the COVID-19 health survey, 18% reported that they had not confirmed their positive test result. Also, people presenting COVID-like symptoms can access testing free of charge without mentioning a previous positive self-test. Anecdotal evidence appears to indicate that this proportion might have further increased, and that confirmation is mostly asked when a sick leave certificate is needed. Thus, the number of reported positive STs is also likely an underestimate. Also, the ratio of reported positive STs over the number of sold tests is lower than the positivity rate reported by survey respondents, supporting the underreporting hypothesis. Still, in the period 17 January – 3 July 2022, 18% of all confirmed cases originated from a positive self-test. Hence, STs appear to have had an important share in detecting COVID-19 cases, at least from January 2022 onwards. The most plausible reason is that starting then, high-risk contacts and travelers who came from high-risk areas were no longer systematically tested. This significantly decreased the number of provider-administered tests and likely increased the use of self-testing after a high-risk contact.

We found that both the number of STs sold, and the number of positive STs followed largely the same trend as provider testing, with increases during periods when the incidence of COVID-19 was highest. We do not know why people were more likely to self-test during those periods, but the data from the two surveys of a representative sample of the adult population both point toward frequent use when they were symptomatic or had a high-risk contact. This was consistent with anecdotal information from health professionals and the findings of another online study of Belgian citizens conducted in early December 2021 [9]. In that study, more than 56% of the participants had taken a self-test or had a family member who had used one. Of the participants who had not yet used a self-test, 61% reported that they had not yet experienced symptoms that prompted them to use one. Thus, it is hypothesized that people are more likely to use a self-test during waves of high incidence because more people experience symptoms and have high-risk contacts. This is further corroborated by the equal trend in the number of sold STs and the trends in number of provider-administered tests in symptomatic people and high-risk contacts, and not for other indications. While it is possible that people self-test more during the waves for the formal indications, we consider it unlikely that this would cause such an increase. The relatively high percentage of positive STs reported by survey participants (17% and 7%) also seem to indicate use for indications with a higher probability of infection than the formal self-test indications. The ratio of reported positive STs over the number of sold tests (on average 0.04) is much lower than the positivity rates in provider-tested symptomatic people and high-risk contacts (on average 26% and 14%, respectively). However, this could be a result of the underreporting of positive STs.

We can therefore conclude that the Belgian government’s guidelines, which explicitly emphasized that self-testing should not be used when having symptoms, and initially also not after a high-risk contact, were not followed. Why people used their own judgement rather than the guidelines we do not know. We can only assume it is a combination of easier access/time savings and unfamiliarity with the guidelines. Which of these factors is predominant requires further investigation.

If and how this affected the isolation and contact tracing strategy, and thereby the control of the epidemic, remains unknown. On the one hand, self-testing comes at the cost of loss of accuracy. Several studies have shown that provider-administered RATs are less sensitive than RT-PCR, particularly when the viral load is low [10–13]. Moreover, studies with point-of-care tests for other infectious diseases already highlighted the importance of trained staff [14–16]. The few studies that have assessed the accuracy of self-administered RATs have indeed shown further decreases in sensitivity [17–20]. Furthermore, while the specificity of RATs is high, it is less than 100%, resulting in a low positive predictive value when the positivity rate is very low [21–25]. For example, with a specificity of 99.5% and a sensitivity of 85%, the positive predictive value is only 77.6% at a prevalence of 2.0%. Another disadvantage is the difficulty in reliable reporting of the results of STs.

The rationale for the restricted self-test indications was indeed a concern for false-negative results due to the lower sensitivity of STs, and false positive results, especially in a context of low virus circulation. If a false-negative self-test result would prevent people with symptoms or high-risk contacts from seeking formal testing, positive cases would go undetected. This would then negatively impact isolation and contact tracing strategies and interfere with epidemic monitoring. These concerns were also shared by international agencies [26]. In people without COVID-symptoms, a false-negative result could lead to abandoning of preventive measures, such as physical distancing and mask-wearing, and thus infection of others. Indeed, in the APB study, a significant proportion of respondents were found not to know how to behave after a negative test result. This is consistent with other international studies that showed that at-home COVID-19 self-test kit users may not follow the recommendations when they test negative. In a randomized trial in the US, for example, 33% of people with a high pre-test probability (having symptoms or after a high-risk contact) said they would not quarantine if the self-test were negative, while this was recommended by the authorities [27].

It can also be speculated that people who tested positive with a self-test and did not report it, were less compliant with the isolation requirement than those who were provider-tested. However, the results of the APB survey seem to indicate that people are well aware that they should isolate if the self-test is positive, as other research has also shown [27].

On the other hand, self-testing has also some major benefits and the use of STs when having symptoms or after a high-risk contact might have had a positive effect as well [28–30]. First, self-testing lowers the threshold for getting tested, a threshold that has shown to be often high. For example, an analysis of the data of the first COVID-19 wave in France estimated that only 31% of individuals with COVID-19-like symptoms consulted a doctor [31]. This is confirmed by seroprevalence studies that show that a high percentage of people with antibodies against SARS-CoV-2 (including when having had COVID-like symptoms) report not having consulted a health care provider or having been tested [32, 33]. Many of the people who self-tested might not have been tested at all if STs had not been available.

Second, STs provide an immediate result and, if testing positive, people will thus isolate sooner. Furthermore, the reduced sensitivity is less a problem in people with a recent onset of symptoms when viral load is high. Several countries expanded therefore their indications for self-testing in late 2021/early 2022 to include when one has (mild) symptoms [34]. Reduced sensitivity is important for high-risk contacts who do not have symptoms and in whom the false negative rate is higher. The period when self-testing was widely used and high-risk contacts had to be tested with PCR was, however, relatively short (November 2021-March 2022).

It is thus possible that the benefits of reduced barriers to testing and a faster result outweigh the risks of reduced sensitivity. In addition, self-testing has also important societal benefits, such as a low cost and relieving overburdened health providers from collecting specimens.

Our evaluation has several limitations. Online surveys have a substantial risk of selection and reporting bias. The survey by DayOne used an existing database of a representative sample of Belgian adults and we believe this minimizes potential selection bias. The Sciensano COVID-19 health surveys were non-probability web surveys because the data were needed quickly. The survey samples were therefore prone to biased estimates as they relied on self-selection and excluded people without internet access or skills. However, Sciensano minimized this bias by setting up partnerships with trustworthy organizations, using a diverse recruitment strategy including multiple platforms to reach different subsets of the population, and assessing the results after every survey and making extra efforts during the next survey when realizing that some population groups were not enough represented. In addition, post-stratification weights were applied in the analysis to partly correct for the sampling bias. Both surveys used closed-ended questions with a fixed order of response options which might have biased the results through primacy effects. However, we do not believe that these potential biases can fully explain the high proportion of using STs when symptomatic or after a high-risk contact. The similarity in the responses between the two surveys further strengthens our conclusion that these were indeed the main reason why people use STs. We did not have data on the number of STs used and the number of negative results and had to use proxies. Nevertheless, we do believe that the bias introduced by these proxies was consistent over time and that it therefore did not have an effect on the trends.

## Conclusion

Since the Delta wave in October-November 2021, self-testing plays an important role and makes up a large part of the testing in Belgium. However, it appears to be mostly used for indications they were not intended for, such as when having symptoms. If this affected the control of the epidemic, we do not know. The lesser sensitivity of self-tests, compared to provider-administered tests, and possibly lower compliance with post-test result measures may have led to additional infections. But this might have been outweighed by the lower threshold for getting tested and getting the result faster. Governments must be aware that, despite proper communication, people will often use their own judgement to decide when and how to use COVID-19 self-tests. Guidelines should therefore be more reality-based and focus on informing and empowering people to manage their own risks, rather than giving them strict indications.

## Data Availability

The datasets used and/or analysed during the current study are available from the corresponding author on reasonable request.

## List of abbreviations

APB: Association of Pharmacists Belgium
HRC: High-risk contact
PCR: Polymerase Chain Reaction
RAT: Rapid Antigen Test
